# Racial, Gender, and Age Dynamics in Michigan’s Urban and Rural Farmers Markets: Reducing Food Insecurity, and the Impacts of a Pandemic

**DOI:** 10.1177/00027642211013387

**Published:** 2021-05-08

**Authors:** Dorceta E. Taylor, Alliyah Lusuegro, Victoria Loong, Alexis Cambridge, Claire Nichols, Maeghen Goode, Ember McCoy, Socorro M. Daupan, M’Lis Bartlett, Erin Noel, Brayden Pollvogt

**Affiliations:** 1 Yale University, New Haven, CT, USA; 2 Ocean Conservancy, Washington, DC, USA; 3 We The People of Detroit, Detroit, MI, USA; 4 University of Miami, Coral Gables, FL, USA; 5 Arizona State University, Tempe, AZ, USA; 6 University of Michigan, Ann Arbor, MI, USA; 7 University of Massachusetts–Boston, Boston, MA, USA; 8 Valparaiso University, Valparaiso, IN, USA

**Keywords:** low income, people of color, White, Black, Latinx, funding, manager, nutrition

## Abstract

In recent decades, the number of farmers markets has increased dramatically across the country. Though farmers markets have been described as White spaces, they can play important roles in reducing food insecurity. It is particularly true in Michigan, where farmers markets were crucial collaborators in pioneering programs such as Double-Up Food Bucks that help low-income residents and people of color gain access to fresh, healthy, locally grown food. This article examines the questions: (1) What are the demographic characteristics of farmers market managers, vendors, and customers? (2) How do these influence market activities? (3) To what extent do farmers markets participate in programs to reduce food insecurity? (4) To what extent do farmers markets serve low-income residents and people of color? And (5) How has the Coronavirus Pandemic (COVID-19) affected farmers’ markets? This article discusses the findings of a 2020 study that examined the extent to which Michigan’s farmers markets served low-income customers and people of color, and participated in food assistance programs. The study examined 79 farmers markets and found that 87.3% of the farmers market managers are White. On average, roughly 79% of the markets’ vendors are White, and almost 18% are people of color. Most of the vendors in the markets participate in nutrition assistance programs. Market managers estimate that about 76% of their customers are White, and about 23% are people of color. Farmers markets operated by people of color attract more customers and vendors of color than those administered by White market managers. Almost half of the farmers markets started operations later than usual in 2020 because of the pandemic. More than a third of the markets reported that their funding declined during the pandemic. Moreover, the number of vendors fell at two-thirds of the markets; customers dipped by more than 40%. On the other hand, the number of people requesting food assistance during the pandemic increased in more than half of the markets.

## Introduction

Farmers markets are an enduring part of the American landscape. Though their popularity has waxed and waned over time, there has been a rapid increase in the number of farmers markets nationwide in recent decades. There were 1,755 farmers markets in 1994; 6 years later, that number reached 2,863. Despite the closure of some markets, in 2019 the National Agricultural Statistics Service identified 8,140 farmers markets nationwide (U.S. Department of Agriculture [USDA], 2020, 2015). The overall growth of farmers markets is attributable to several factors. These include burgeoning consumer interest, increased government assistance, greater support for local farmers, and organizing by local farmers. Other factors influencing growth are the provision of increased access to freshly produced food through direct-to-consumer sales, increased social interactions, and a greater sense of community in neighborhoods and cities (Hunt, 2007; Kahin et al., 2017; Brown & Miller, 2008; Oberholtzer & Grow, 2003).

As the number of farmers markets grows, so does the economic activity they generate in local communities. In 1997, farmers markets generated $551 million in sales. However, by 2015, farmers markets accounted for 23% of direct-to-consumer sales (Martinez et al., 2010; USDA, 2016). According to the USDA, in 2020 farmers markets and other direct-to-consumer sales contributed roughly $9 billion to the economy (Perdue, 2020).

Like the rest of the country, the number of farmers markets in Michigan is rising. There were about 90 farmers markets in the state in 2001. That number grew to about 300 by 2016 (Gagliardi et al., 2016; Michigan Farmers Market Association [MIFMA], 2020). This article presents the findings of a study of farmers markets in Michigan. Michigan is a logical place to study farmers markets as the Agricultural Marketing Service indicates that such markets are concentrated in the Northeast, Midwest, and the West Coast (Martinez et al., 2010). Additionally, Michigan’s $132 million in direct-to-consumer farm sales is the seventh highest in the country (USDA, 2016). The article examines four questions: (1) What are the demographic characteristics of the farmers market managers, vendors, and customers, and how do these influence market activities? (2) To what extent do farmers markets participate in programs to reduce food insecurity? (3) To what extent do farmers markets serve low-income residents and people of color? and (4) How has the Coronavirus Pandemic (COVID-19) affected the operations of farmers markets?

## Literature Review

### Farmers Markets as White Spaces

One of the dominant narratives about farmers markets is the depiction of the markets as White spaces. In this vein, Allen (2004) and Payne (2002) argue that White organic farmers are the dominant vendors at California’s farmers markets. Several studies find that the patrons of farmers markets are also overwhelmingly White (Byker et al., 2012; Conner et al., 2010; Elepua et al., 2010; Onianwa et al., 2006). Some scholars conclude that the presence of primarily White vendors and customers in farmers markets gives the appearance of a racialized White space (Hoover, 2013; Kobayashi & Peake, 2000; Lambert-Pennington & Hicks, 2016; Saldanha, 2006; Slocum, 2007).

Alkon and McCullen (2010) argue further that farmers markets attract affluent and liberal customers. Despite these findings, farmers market managers tend not to perceive the markets as racialized spaces (Alkon & McCullen, 2010). Other researchers such as Lambert-Pennington and Hicks (2016) also portray farmers markets as White spaces catering to affluent, privileged Whites. Slocum (2007) contends that the markets reflect White privilege.

But this line of argument runs the risk of rendering invisible farmers markets that operate in people of color communities. Thus, it is also essential to recognize that farmers markets can and do thrive in low income, communities of color. Researchers such as Ruelas et al. (2012) and Onianwa et al. (2006) have documented farmers markets in Latinx and Black communities. Indeed, one of the oldest farmers markets in the country, Eastern Market, is in Detroit (Johnson & Thomas, 2005). The Flint Farmers’ Market is also more than a century old (Morckel & Colasanti, 2018).

### Characteristics of Farmers Market Managers

Why the interest in farmers market managers? Managers are key actors and influential decision-makers in farmers markets and other segments of alternative food networks. Farmers market managers play vital roles in the social construction of discourses about food, consumption, and spaces that sell locally grown and organic food. It is the case because the managers make critical decisions about market policies; operations; programming; strategic thinking and positioning; the sociocultural framing and manifestation of the space; racial, class, and gender dynamics in markets; and community engagement and outreach.

Thirty-one percent of all farms have a female manager. However, of the roughly 300,000 farm managers who sold direct-to-consumers nationwide in 2015, 38% are women (USDA, 2016). Those who manage farmers markets are a subset of this group. Researchers studying farmers market managers have found that such staff usually identified themselves as farmers. They also found that the managers were younger and more educated than other farmers in their state and region (Hunt, 2007). Others such as C. L. Miller and McCole (2014) also found that farmers market managers were younger and more educated than other farmers. Their survey of farmers and farmers market managers found that 25.7% of the farmers market managers were less than 40 years of age. In comparison, 16.2% of the farmers surveyed were less than 40 years old. While 72.7% of the farmers market managers were college graduates or had advanced degrees, only 47.3% of other farmers were similarly educated.

A study of 38 farmers market managers in Texas and Arkansas found that 82% were women (Mohammad et al., 2019). The MIFMA studied 108 market managers and found that 81% of the market managers were women; however, men managers received higher compensation for managing markets than women market managers (Smalley et al., 2017). C. L. Miller and McCole’s (2014) study of 33 farmers market managers in southeast Michigan also found that women dominate the market managers’ position—87.5% were women.

C. L. Miller and McCole (2014) found that 54.9% of the market managers studied were 50 years or older. Similarly, about 59% of the respondents in the MIFMA study said they were 50 years or older. Two thirds of the managers had managed markets for five years or less (Smalley et al., 2017). Other studies have found farmers market managers to have less farming experience than other farmers (Martinez et al., 2010). Data from the USDA also bolster this finding. The department reports that 77% of farms with direct-to-consumer sales and 78% of all farms nationwide are managed by farmers with ten or more years of farming experience (USDA, 2016).

### Characteristics of Farmers Market Vendors

Few studies examine the characteristics of farmers market vendors. In one such study, Hunt (2007) studied 81 farmers market vendors in Maine and found that the farmers market vendors tended to be younger than other farmers in the state. While the mean age for the farmers market vendors was 44 years, the mean age of Maine’s farmers was 54 years. Farmers who were market vendors were more highly educated than other Maine farmers. While 53% of the farmers market vendors had a college degree, only 19% of the remaining farmers in the state were similarly educated.

A study of 125 South Carolina farmers market vendors found 57% operated men-owned businesses at the markets (Campbell, 2014). Conversely, the gender profile of farmers market vendors in Texas and Arkansas differs from South Carolina. Sixty-two percent of the 85 farmers market vendors in Texas and Arkansas were women (Mohammad et al., 2019).

The South Carolina study found that 71.3% of the vendors were White, 13.9% were Black, and 5.6% were Latinx and Asian. On average, vendors sold produce at their primary farmers markets for 4.4 years (Campbell, 2014). Researchers also studied farmers market vendors in West Virginia and found that 61.8% of the 183 vendors were 50 years or older. The study also found that 57.9% of the vendors were men and 26.2% were women; the remaining vendors were in partnership groups. About 53% of the vendors were college educated (S. M. Miller, 2005).

### Characteristics of Farmers Market Customers

Race and ethnicity are associated with access to food. Studies find that communities of color, particularly Black and Latinx, have limited access to food outlets that sell fresh and healthy foods (Taylor & Ard, 2015; Singleton et al., 2020; Zenk et al., 2014). As a result, farmers markets are characterized as food outlets that can help to enhance food access to residents in poor communities and communities of color.

But some researchers question whether demographic characteristics can predict purchasing behavior. For instance, a study of consumers who purchase locally produced food in the southeastern United States found that demographic characteristics were poor predictors of buying local dairy products (Best & Wolfe, 2009). Similarly, two national studies found that people of various education and income levels purchase locally produced food (Keeling-Bond et al., 2009; Zepeda & Li, 2006). Wolf et al. (2005) studied 336 farmers market patrons in San Luis Obispo County, California reported mixed results. The researchers found that the age, income levels, and occupational status of farmers market customers and shoppers who do not patronize farmers markets were similar. However, the researchers found that farmers market customers tended to be women, married, and had higher educational attainment. Hence, 64% of the farmers market customers are women, 61% are married, and 89% have a college or graduate school education. Half of the study participants who did not go to farmers markets, were women, 46% were married, and 79% had attended college or graduate school.

Several studies of farmers markets find a relationship between gender and shopping at such markets; they find that women comprise between 64% and 77% of the patrons of these food outlets (Baker et al., 2009; Byker et al., 2012; Elepu & Mazzocco, 2009; Zepeda, 2009). Onianwa et al. (2006) found that 72% of the 222 farmers market patrons surveyed in Huntsville and Birmingham, Alabama were women and 49% were White. The researchers also found that 80.2% of the customers had more than a high school education, 70.3% were married, and 90% had household incomes of $25,000 or more.

Similarly, Elepu and Mazzocco (2009) found that 76.7% of 379 farmers markets patrons studied in Chicago and East St. Louis were women. The patrons were predominantly White—82.9% were White, 10.7% were Black, 2.6% Asian, and 0.2% were Native American. Moreover, 93.6% were college educated (43.7% have a graduate school education). Farmers markets customers also tend to have professional jobs; 41.8% do. The study also found that 43.2% earn $75,000 or more. The customers in these farmers markets were older; 23% were 35 to 44 years old, and another 54.5% were 45 years or older. A study of 405 customers of the Flint Farmers’ Market in Michigan found that 77% of the participants were women, and 71% were 45 years or older (Sadler et al., 2013).

Ruelas et al. (2012) surveyed 415 farmers market patrons in East Los Angeles and 1,374 in South Los Angeles and found that most respondents were low income Latinx women. The researchers found that 93% of the East Los Angeles farmers market customers were Latinx; so were 78% of the South Los Angeles farmers market patrons. The percentages of women shoppers at each market were 88% and 80%, respectively. About 55% of the customers of both markets had less than 12 years of education and earned less than $15,000 annually.

Other scholars also find that farmers market customers tend to be older (more than 40 years old), highly educated, have higher income, are employed, are urban dwellers, and are women. Farmers market customers also tend to belong to environmental groups (Brooker & Eastwood, 1989; Brown, 2003; Byker et al., 2012; Conner et al., 2010; Eastwood, 1996; Eastwood et al., 1999; Govindasamy et al., 1998).

Researchers studying low-income customers in Illinois’s Link Match program that provides monetary incentives for Supplemental Nutrition Assistance Program (SNAP) recipients to purchase food at farmers markets found that Blacks shopped at farmers markets infrequently. Blacks shopped at the markets once monthly or less (Singleton et at., 2020). The farmers markets are also devoid of the low-income, low-wage Latinx and other people or workers who labor on the farms (Alkon & McCullen, 2010).

### Farmers Markets and Food Insecurity

Farmers markets have been publicized as places that can enhance access to healthy foods and increase the consumption of locally grown produce for low-income residents and communities of color. It is particularly true in communities that lack food stores that sell healthy foods. Farmers markets can serve the poor and reduce food insecurity by participating in government initiatives such as the SNAP and the Women, Infant, and Children (WIC) programs (Becot et al., 2020; Karpyn et al., 2012; Widener et al., 2012).

Between 2012 and 2017, the number of SNAP recipients who shop at farmers markets increased by 35.2%. In 2017, farmers markets obtained more than $22.4 million from the purchases of SNAP recipients (Farmers Market Coalition, 2020). In 2019, 4,076 or 50.1% of the farmers markets in the country participated in some form of federal nutrition programs such as SNAP, WIC, or the Senior Farmers Market Nutrition Program (SFMNP). Moreover, 55.9% of the farmers market vendors accept SNAP, 66.7% accept WIC, and 66.3% accept SFMNP (USDA, 2020).

To this end, there are a variety of initiatives that lower the cost of food sold at farmers markets. The lower price removes one of the barriers that hinder poor people from shopping at farmers markets. Such programs encourage and incentivize low-income families to redeem food assistance benefits at farmers markets (Baronberg et al., 2013; Becot et al., 2020; Buttenheim et al., 2012; Cole et al., 2013; Engel & Ruder, 2020; Jilcott Pitts et al., 2015; Lindsay et al., 2013; Young et al., 2013).

The Fair Food Network introduced Double Up Food Bucks (DUFB) in Michigan with pilot programs in five farmers markets in Detroit in 2009. The DUFB provided SNAP recipients with a one-to-one matching credit of up to $20 on each market day for each dollar spent on SNAP-eligible items, and SNAP customers can use the credit to purchase Michigan-grown produce. The program quickly expanded to 250 farmers markets in Michigan. In 2019, the program, which now operates at 615 farmers markets and 313 grocery and corner stores in 28 states, accounts for $15.2 million in SNAP and DUFB sales. The program served almost 222,000 families (443,424 individuals) in 2019 (Fair Food Network, 2020; Pasquale et al., 2019). Studies show that DUFB increases the purchase of vegetables from farmers markets and the intake of such vegetables (Young et al., 2011; Savoie-Roskos et al., 2016).

Garner et al. (2020) studied DUFB programs in Utah and New York and found that program participants were satisfied that farmers market produce was more affordable. However, study participants reported a lack of information about the program and that it was inconvenient to access. In Mino et al.’s (2018) study, farmers market managers in Michigan reported that customers and vendors found the rules governing the various food assistance programs confusing. The confusion is most apparent at the start of the market season.

The programs described above also serve the additional function of increasing sales and providing income to farmers at a time when farmers markets are nearing or have reached saturation nationwide (Low et al., 2015). A study of New York City’s Health Bucks Program—which offers a $2 coupon to SNAP recipients for every $5 spent at farmers markets—found that SNAP recipients spent more money at farmers markets that participate in the program than those markets that did not participate in the program (Baronberg et al., 2013). San Diego’s Fresh Fund program assessment found that 48% of the farmers markets revenues came from customers participating in the incentive program (Lindsay et al., 2013).

### Interrogating Farmers Markets

Studies probing the notion of farmers markets as White spaces could be more nuanced. Some researchers working with the framework of White spaces rely on assessments of a few markets (Alkon & McCullen, 2010; Kobayashi & Peake, 2000; Lambert-Pennington & Hicks, 2016; Saldanha, 2006; Slocum, 2006; 2007; 2008; Guthman 2008a; 2008b). For instance, though Alkon and McCullen (2010) rely heavily on this concept, they study only two farmers markets and briefly describe a third. Lambert-Pennington and Hicks (2016) study only one farmers market. We contend that researchers should examine the concept of White spaces in larger samples of farmers markets in urban and rural markets. This approach could allow for comparisons and discovery of different dynamics that influence if and how farmers are racialized. Researchers should also recognize that factors such as the demographic characteristics of farmers market managers, staff, and volunteers, market locations, and market engagement with food assistance initiatives, are relevant to the discussion of the racial/ethnic configuration of the market space. Other factors such as gender and age are also applicable in this type of research. Despite the widespread use of the idea of the Whiteness of farmers markets, researchers have been slow to analyze and compare farmers markets that are managed by people of color with those operated by Whites.

Though farmers markets are framed as places where the poor can purchase fresh and healthy foods at a reasonable price by participating in food subsidy programs, most low-income residents do not obtain their food at farmers markets. Despite efforts to increase the number of low-income people shopping at farmers markets, less than 1% of food assistance dollars are redeemed at farmers markets (Roubal et al., 2016).

A study of 51 SNAP recipients in New Orleans found that only 47% of the study participants had ever been to a farmers market. The study found that 88% of the respondents shopped for fruits and vegetables at grocery stores and 84% obtained these items at Walmart (Nuss et al., 2017). Skizim et al. (2017) found that 63% of low-income New Orleans residents never visited a farmers market, while 27% of middle- and high-income residents never went to farmers markets.

This experience goes well beyond New Orleans. By and large, farmers markets provide limited opportunities for poor people to shop for food. An estimated two-thirds of low-income residents buying foods purchase them from grocery stores and other food outlets. This being the case, the Fair Food Network expanded the use of DUFB beyond farmers markets to include brick and mortar food stores (Fair Food Network, 2020; Pasquale et al., 2019).

Price is a barrier that prohibits some from shopping at farmers markets (Bond et al., 2006; Grace et al., 2007; Jilcott Pitts et al., 2010; Onianwa et al., 2006; Wetherill & Gray, 2015; Wolf et al., 2005). A study of 143 SNAP customers at farmers markets found that only 35.9% strongly agreed that they could afford to purchase the fruits and vegetables at the market. These market patrons strongly agreed that fruits and vegetables were essential to their health (Amaro & Roberts, 2017).

The above studies reveal a glaring blind spot in the approaches that researchers rely on to investigate farmers markets and low-income access to food. Most of the studies examine the use of SNAP, WIC, DUFB, and other nutrition assistance programs to measure the role farmers markets play in reducing food insecurity. These studies seek to understand how programs are designed to get poor people to go to farmers’ markets and purchase food. Thus far, researchers have shied away from examining other ways farmers markets can reduce food insecurity that does not involve measuring how much poor people spend in the markets. Market activities that go understudied include partnerships in which farmers markets donate food to community organizations such as emergency food assistance organizations, schools, and churches, or donating food to people who request it.

Distance and inconvenient location can be a deterrent to shopping at farmers markets. Farmers market patrons report traveling from 6 to 17 miles to reach a market (Byker et al., 2012; Hunt, 2007; Jilcott Pitts et al., 2010; Onianwa et al., 2006; Varner & Otto, 2008). Latinx residents of East and South Los Angeles said they traveled 4 miles to reach local farmers markets (Ruelas et al., 2012). Fifty-six percent of the Flint Farmers’ Market patrons reported that they travel less than 6.2 miles to get to the market (Sadler et al., 2013).

Latinx respondents in a Michigan study reported that they place a high value on farmers markets but refrained from shopping in them because they did not feel welcomed. Language and other cultural barriers also discourage Latinx and other people of color from shopping at farmers markets (Conner et al., 2010).

Farmers market managers report that they experience significant burdens when administering food assistance programs even when they have substantial organizational infrastructures. In particular, the processing of food assistance transactions consumes a great deal of time. Market managers worry that the programs will be unsustainable over the long haul because of the magnitude of resources needed to support such programming (Mino et al., 2018).

The findings presented below come from a study that examines a robust sample of farmers markets in different geographic locales—rural areas, small towns, and urbanized areas—in Michigan. It is one of the first studies to take this comparative approach. The study also examines demographic variables related to farmers market managers, vendors, and customers. It assesses market participation in food assistance programs and other activities that help reduce food insecurity. Here again, the study breaks new ground. It goes beyond the typical government-run nutrition assistance program assessment to explore a broader range of market activities to reduce food insecurity. It is also one of the first studies to examine the impacts of the COVID-19 pandemic on market operations and food insecurity.

## The Michigan Context

Michigan, a state with 9,986,857 residents in 2019, has a growing population. Women comprise 50.7% of the population, and 21.5% are younger than 18 years. Michigan is a predominantly White state. Whites (not Hispanic) constitute 74.7% of the population, and Blacks comprise 14.1%, Latinx 5.3%, Asians 3.4%, and Native Americans make up 0.7% of the population. The state’s median household income is $54,938 in 2018 dollars. Michigan’s poverty rate of 13% is high (U.S. Census Bureau, 2020a).

**Map 1.**
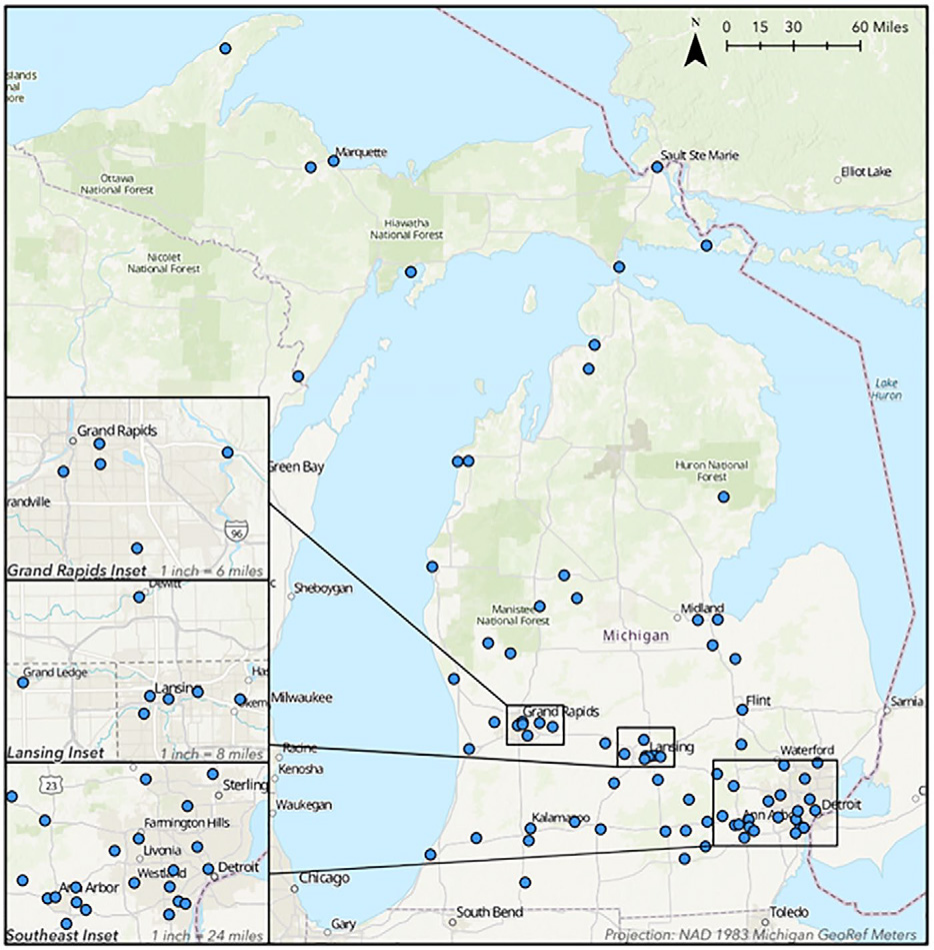
The location of Michigan’s farmers markets contained in the sample.

Blacks, Latinx, and Asians are concentrated in the southern part of the state in the Detroit metropolitan area, Ypsilanti, Flint, Saginaw, Lansing, East Lansing, Grand Rapids, and Benton Harbor (see Map 1). Though Detroit and Flint are among Michigan’s most well-known cities, Benton Harbor has one of the highest percentages of Black residents of any municipality in the state. Benton Harbor—located in the southwestern part of the state—is 85.6% Black, 8.3% White, and 4.9% Latinx. In comparison, Michigan’s largest city, Detroit, is 78.6% Black, 10.3% White, and 7.6% Latinx. On the other hand, Flint is 53.7% Black, 37.4% White, and 3.9% Latinx. Saginaw, a city located about 40 miles north of Flint, is 45% Black, 37.1% White, and 14.4% Latinx. The state’s capital city—Lansing—is 54.9% White, 22.3% Black, 12.5% Latinx, and 3.6% Asian. The adjacent town, East Lansing, is 71.4% White, 12.8% Asian, 7.8% Blacks, and 4.8% Latinx.

Michigan’s second largest city, Grand Rapids, is in the western part of the state. Whites comprise 59.4% of the city’s population, Blacks 19.2%, Latinx 15.9%, and Asians 2.3%. Muskegon is another of Michigan’s western cities, and it has a population that is 53.8% White, 31.3% Black, and 8.6% Latinx. Holland is also on the west side of Michigan. The city is 67.7% White, 22.8% Latinx, 4.5% Black, and 2.7% Asian. Michigan’s Native American population is dispersed across the state. In some areas, such as Chippewa County—in the Upper Peninsula—Native Americans comprise 16.3% of the county’s residents (U.S. Census Bureau, 2020a).

## Methodology

### Definition of Farmers Markets

For the purposes of this study, we define a farmers market as a public gathering of two or more farmers and their representatives at a common, recurrent physical location to sell agricultural products and other items they produce directly to customers. The market may include vendors that market managers and their boards deem appropriate (Farmers Market Coalition, 2020; MIFMA, 2017; USDA, 2020).

### Survey Methodology: Identifying and Selecting Farmers Markets in Michigan

We studied Michigan farmers’ markets during the summer and fall market season in 2020. We used MIFMA’s (2020) directory of farmers markets, the Agricultural Marketing Service (2020) directory, and the Web to identify functioning farmers markets in the state. We communicated with 210 farmers market managers for whom we had contact information to ask them to complete a survey about the farmers market they operate. The survey, designed on a Qualtrics platform, could be administered by telephone or self-administered (Qualtrics, 2020). Market managers were usually too busy during daytime hours to take a telephone survey, so we sent managers a hyperlink to the survey to complete it at their convenience. The survey took about 45 minutes to complete. Each manager who completed the survey was offered $35 compensation for their time. We surveyed from June 27 to November 18, 2020. We received 95 responses, but only 79 of these were usable. The number of usable surveys accounts for 38% of the instruments initially distributed. We downloaded data from the Qualtrics survey into SPSS 27.0 for statistical analysis (IBM, 2020).

### Urbanized Areas, Urban Clusters, and Rural Designations

We used U.S. Census Bureau 2020 guidelines to classify urban and rural areas. According to the census, an urbanized area is a continuously built-up setting with a population of 50,000 or more. The bureau classifies an urban cluster—also known as a small urban area—as a locale outside of a metropolitan area or central city that is incorporated with a population of at least 2,500 residents but less than 50,000 inhabitants. Third, rural areas are incorporated entities or census designated places with fewer than 2,500 inhabitants; these are not located in urbanized areas (U.S. Census Bureau, 2020b; Michigan Department of Transportation, 2013; Ratcliffe et al., 2016).

### Spatial Mapping

First, we used the Census’s Geographic Information Systems Open Data (2020) to identify the urban boundaries of Michigan. The Adjusted Census Urban Boundary layer is a single polygon that represents the boundary of each locality. Next, the SPSS data file was converted to a comma separated value file with farmers market addresses to create maps with the locations of each farmers market studied. We used ArcPro 2.7.1 (ESRI, 2021a) and the ArcGIS World Geocoding Service (ArcGIS Developers, 2021) to geocode the farmers market addresses, turning each address into a point on the map. The points were then projected onto a map using the NAD 1983 Michigan GeoRef projected coordinate system (ESRI, 2021b). Because some of the farmers markets are close, we included inset maps to depict the market locations in Grand Rapids, Lansing, and Southeast Michigan (the Detroit and Ann Arbor-Ypsilanti metropolitan areas.

## Results

### Market Locations

This article analyzes 79 farmers markets. Map 1 shows the location of the markets studied. Most of the markets—80.2%—are members of MIFMA. Most of the farmers markets in the sample are in the southernmost part of the state; this is the most heavily populated part of Michigan. Nonetheless, the sample contains farmers markets located in the northern part of the state’s Lower Peninsula and the Upper Peninsula. Table 1 shows that 38 or almost half of the farmers markets in the study are in urbanized areas. A quarter (19) are in rural areas, and 27.8% (22) are in urban clusters.

**Table 1.** Race/Ethnicity, Gender, and Age of Michigan’s Farmers Market Managers.

Market characteristics	Total sample		Market location			*M*
	Number of markets reporting	Percent	Rural (*n* = 19)	Urban clusters (*n* = 22)	Urbanized areas (*n* = 38)	
Total	79	100.0	24.1	27.8	48.1	
Race or ethnicity of market managers						
White	69	87.3	94.7	90.9	81.6	
Person of color	10	12.7	5.3	9.1	18.4	
Gender of market managers						
Women	58	73.4	57.9	77.3	78.9	
Men	21	26.6	42.1	22.7	21.1	
Age of market managers						
Primary market manager	78					48.2 years
Younger than 40 years	28	35.9	15.8	40.9	43.2	
40-54 years	22	28.2	31.6	22.7	29.7	
55 years and older	28	35.9	52.6	36.4	27.0	
Secondary manager	31					45.1 years
Younger than 40 years	9	29.0	20.0	22.2	35.3	
40-54 years	15	48.4	40.0	55.6	47.1	
55 years and older	7	22.6	40.0	22.2	17.6	

### Demographic Characteristics of the Market Managers

Michigan’s farmers market managers are primarily White women. The study shows that 87.3% of the managers are White, and 73.4% of the markets are managed by individuals or teams comprised solely of women. Though less than 10% of the market managers in rural areas and urban clusters are people of color, 18.4% of the managers in urbanized areas are people of color. Rural farmers markets are much more likely than other locales to have men as market managers. Hence, 42.1% of rural markets have men managers. In contrast, 22.7% of the markets in urban clusters and 21.1% in urbanized areas have men managers.

Seventy-eight of the market managers reported their age. Their mean age is 48.2 years. Thirty-one markets have co-managers, the co-managers are younger than the primary managers. The mean age of the co-managers is 45.1 years. The market managers in urbanized areas tend to be younger than those in rural markets. While 15.8% of the primary market managers in rural markets are less than 40 years of age, more than 40% of those managing markets in urban clusters and in urbanized areas are younger than 40 years. The opposite is true for rural markets. The study found that 52.6% of the rural markets had primary managers who were 55 years or older. For the remainder of this article, when the age of farmers market managers is discussed, the data refer to the age of the primary market manager.

White market managers tend to be older than market managers of color. That is, 30.9% of the White market managers are younger than 40 years, 30.9% are 40 to 54 years old, and 38.2% are 55 years or older. In contrast, 70% of the people of color market managers are less than 40 years old, 10% are 40 to 54 years of age, and 20% are 55 years or older. There are also marked differences between race/ethnicity, age, and localities in which White and people of color market managers operate markets. Overwhelmingly, people of color managers operate farmers markets in urbanized areas. Moreover, the market operators of color, tend to be younger than White market operators. While 36.7% of the White-managed farmers markets in urbanized areas are more youthful than 40 years, 71.4% of the people-of-color managed farmers markets in urbanized areas are operated by managers younger than 40 years.

The article examines three indicators of market size—the number of stalls or booths available for vendors, the number of vending spots that are occupied each week, and the number of customers visiting the market each week (see Table 2). Farmers markets had from 2 to 300 spots for vendors, and markets had as many as 220 vendors occupying stalls weekly. They had a waiting list of as many as 100 vendors. The farmers markets also have from 50 to 300 customers per week.

**Table 2.** Characteristics of Michigan’s Farmers Markets.

Market characteristics	Total sample		Market location			Race/ethnicity of market managers		Gender of market managers		Age of primary market managers		
	Number of markets reporting	*M*	Rural	Urban clusters	Urbanized areas	White	People of color	Women	Men	Younger than 40 years	40 to 54 years	55 years or older
Size of the markets												
Maximum number of vending spots available in market	70	49.1	52.3	35.1	55.4	51.6	27.0	47.8	53.5	49.2	49.4	48.7
Number of vending spots usually occupied weekly	73	30.9	18.9	20.6	42.9	32.2	18.4	32.8	26.0	37.7	29.6	24.8
Number of customers visiting market weekly	71	215.2	189.4	175.6	251.4	214.0	228.3	217.9	208.8	232.6	205.5	204.2
Demand for market space												
Number of potential vendors are on your waiting list	37	9.1	3.8	1.6	13.7	9.5	5.8	7.8	12.7	3.6	14.8	11.0
Longevity and stability												
Mean number of years market has been in current location	79	12.4	10.3	11.2	14.2	13.0	8.3	11.9	14.0	15.6	10.8	10.9
Mean number of years current market manager in position	79	4.4	4.5	4.1	4.5	4.6	2.7	4.6	3.8	2.1	4.9	6.3
Mean age of primary farmers market manager	78	48.2	55.4	47.7	44.7	49.4	40.1	46.8	51.9	33.1	47.9	63.4
Race and ethnicity of vendors												
Mean percentage of white vendors	68	78.8	90.5	78.3	73.8	79.8	70.3	75.0	87.2	85.1	70.3	81.1
Mean percentage of vendors of color	60	17.7	11.4	11.6	23.5	17.2	22.0	18.1	16.8	13.4	24.1	15.3
Race and ethnicity of customers												
Mean percentage of white customers	68	76.2	89.8	74.8	70.4	77.4	21.8	72.7	84.1	76.6	73.4	78.3
Mean percentage of customers of color	67	23.0	10.9	21.4	29.5	65.9	33.9	26.3	15.9	23.4	23.4	21.8
Gender of vendors												
Mean percentage of women vendors	69	56.6	61.8	60.5	51.7	38.4	56.1	57.0	41.1	58.2	52.2	58.9
Mean percentage of men vendors	69	42.4	38.2	40.7	45.5	42.8	60.6	55.6	45.3	44.5	47.7	36.3
Gender of customers												
Mean percentage of women customers	67	60.3	57.2	58.6	62.7	59.9	63.1	60.2	60.5	58.7	60.5	61.9
Mean percentage of men customers	67	39.3	42.6	41.0	36.8	37.1	39.6	39.3	39.3	40.0	39.9	37.8
Hours of operation												
Number of hours operated per week during the spring	68	5.3	3.8	5.3	6.1	5.4	4.3	5.5	4.7	4.7	6.8	4.7
Number of hours operated per week during the summer	77	8.1	4.6	9.2	9.1	7.9	9.5	9.0	5.7	8.4	7.9	8.0
Number of hours operated per week during the fall	72	7.0	4.5	5.8	8.9	6.9	8.1	7.7	5.2	8.0	7.5	5.8
Number of hours operated per week during the winter	31	5.3	2.5	3.7	6.7	5.3	5.3	5.1	6.2	4.5	7.9	3.9

The markets in urbanized areas led on all indicators of size. Urbanized markets had the largest number of vending spots available (x̄= 55.4) and the highest number of vending spots occupied weekly (x̄= 42.9). Markets in urbanized areas had more than twice as many vendors as the rural markets and farmers markets in urban clusters. The rural markets tended to have the largest number of vacancies. While rural markets had approximately 33.4 vacant vendor spots each week, urban clusters had about 14.5 vacancies, and markets in urbanized areas had a mean of 12.5 spots open weekly. Markets in urbanized areas also outpaced rural markets and those in urban clusters regarding the number of customers visiting the market. On average, 251.4 customers visit markets in urbanized locales. In comparison, 189.4 visit rural markets, and markets in urban clusters get 175.6 customers per week. Markets in urbanized areas also have a much longer waiting list for vendors to obtain a spot than the markets in urban clusters or rural areas.

White farmers market managers operate markets that are significantly larger than the ones that people of color run. While White managers have a mean of 51.6 vendor slots in their markets and have an average of 32.2 of those occupied each week, the markets that people of color operate have a mean of 27 vendor spots and a mean of 18.4 of those stalls are occupied weekly. However, the people of color managers oversee markets that have more customers. A mean of 228.3 people visit the markets with a person of color manager compared to a mean of 214 people visiting the markets with White managers.

The locations at which Michigan’s farmers markets operate are very stable. On average the markets have operated in the exact location for about 12.4 years. The markets in urbanized areas have operated in their current location longer than markets in small towns or rural areas. But farmers market managers have relatively short tenure in the job. The mean number of years the managers have been operating their current market is 4.4 years. The tenure is similar across the three types of locales studied. However, the people of color managers are not only the youngest; they have been on the job for the shortest length of time. The average tenure of a person of color market manager is 2.7 years.

Rural farmers markets have substantially higher percentages of White vendors and customers than other farmers markets. Farmers markets operated by people of color tend to attract more people of color vendors and customers than White-run markets. Twenty-two percent of the vendors and 33.9% of the customers in people-of-color-managed farmers markets are ethnic and racial minorities.

In general, the youngest managers—those younger than 40—tend to manage markets with the lowest vendor vacancy rates and the largest number of customers per week. These markets also tend to have the shortest waiting list for vendors. However, the youngest managers also have the shortest tenure at their markets; they have been managing their current markets for a mean of 2.1 years. The markets with the youngest managers tend to have the lowest number of vendors of color, but markets with the youngest managers have slightly more customers of color than markets run by the oldest managers.

### Participation in Programs to Reduce Food Insecurity

Michigan’s farmers markets participate in various programs to reduce food insecurity. The study assessed participation in six types of subsidized nutrition programs, viz., DUFB, SNAP (Bridge Card), WIC, Project FRESH, Hoop Houses for Health, and Food Navigator. The study found that roughly two thirds of the vendors in the markets sampled participated in DUFB and SNAP and 63.8% of the vendors accepted WIC (Table 3). Though less well known than the three preceding programs, 71.8% of the vendors participated in Project FRESH. However, less than 20% of the market vendors participated in the Hoop Houses for Health or Food Navigator programs. Food assistance benefits are processed through an electronic benefits transfer (EBT) card reader that farmers markets install. The EBT card that nutrition assistance program participants use to make food purchases in Michigan is commonly referred to as the Bridge Card.

**Table 3.** Farmers Market Vendors and their Participation in Nutrition Assistance Programs.

Nutrition assistance programs	Total sample	Market location			Race/ethnicity of market managers		Gender of market managers		Age of primary market managers		
	Number of markets reporting	Rural	Urban clusters	Urbanized areas	White	People of color	Women	Men	Younger than 40 years	40 to 54 years	55 Years or older
Amount of market vendors who participate in Double Up Food Bucks	71	18	18	35	64	7	51	20	23	22	25
Percent of vendors who do not participate in the program	29.6	33.3	50.0	17.1	29.7	28.6	31.4	25.0	26.1	22.7	40.0
Percent of vendors who participate in the program	66.2	63.9	50.0	80.0	65.6	71.4	66.6	65.0	69.6	72.8	56.0
Don’t know	4.2	2.8	0.0	2.9	4.7	0.0	2.0	10.0	4.3	4.5	4.0
Amount of market vendors who participate in the Supplemental Nutrition Assistance Program (SNAP)	70	18	17	35	63	7	50	20	23	22	24
Percentage of vendors who do not participate in the program	30.0	33.3	47.1	20.0	30.2	28.6	34.0	20.0	21.7	22.7	45.8
Percentage of vendors who participate in the program	67.1	61.1	52.9	77.1	66.6	71.4	66.0	70.0	78.3	72.8	50.0
Don’t know	2.9	5.6	0.0	2.9	3.2	0.0	0.0	10.0	0.0	4.5	4.2
Amount of market vendors who participate in the Women, Infant, and Children (WIC) Program	69	16	18	35	62	7	51	18	23	21	24
Percentage of vendors who do not participate in the program	36.2	31.3	50.0	31.4	35.5	42.9	39.2	27.8	34.8	33.3	41.7
Percentage of vendors who participate in the program	63.8	68.7	50.0	68.6	64.5	57.1	60.8	72.2	65.2	66.7	58.3
Don’t know											
Amount of market vendors who participate in Project FRESH	71	18	18	35	64	7	51	20	23	22	25
Percentage of vendors who do not participate in the program	28.2	27.8	38.9	22.9	26.6	42.9	29.4	25.0	21.7	27.3	36.0
Percentage of vendors who participate in the program	71.8	72.2	61.1	77.2	73.4	57.1	70.6	75.0	78.3	72.7	64.0
Don’t know											
Amount of market vendors who participate in Hoop Houses for Health	63	15	17	31	56	7	47	16	21	20	21
Percentage of vendors who do not participate in the program	74.6	60.0	88.2	74.2	76.8	57.1	72.3	81.3	71.4	75.0	76.2
Percentage of vendors who participate in the program	11.1	13.3	0.0	16.1	10.7	14.3	12.8	6.2	14.3	10.0	9.5
Don’t know	14.3	26.7	11.8	9.7	12.5	28.6	14.9	12.5	14.3	15.0	14.3
Amount of market vendors who participate in Food Navigator	63	15	17	31	56	7	47	16	21	20	21
Percentage of vendors who do not participate in the program	71.4	66.7	82.4	67.7	71.4	71.4	72.3	68.8	71.4	70.0	71.4
Percentage of vendors who participate in the program	15.9	6.6	5.8	25.8	16.1	14.3	19.2	6.2	14.3	20.0	14.3
Don’t know	12.7	26.7	11.8	6.5	12.5	14.3	8.5	25.0	14.3	10.0	14.3
Amount of market vendors who participate in Prescription for Health	63	15	17	32	57	7	46	18	22	21	20
Percent of vendors who do not participate in the program	59.4	60.0	70.6	53.1	61.4	42.9	61.1	61.1	45.5	61.9	70.0
Percentage of vendors who participate in the program	29.7	20.0	17.6	40.6	28.1	42.8	22.2	30.2	40.9	28.6	20.0
Don’t know	10.9	20.0	11.8	6.3	10.5	14.3	16.7	8.7	13.6	9.5	10.0

Market vendors in urbanized areas were more likely to participate in DUBF, SNAP, and Project FRESH than vendors elsewhere. That is, 80% of those selling at markets in urbanized areas accepted DUFB, and 77% participated in SNAP and Project FRESH. In contrast, only half of the vendors selling at farmers markets in urban clusters accepted DUFB and WIC.

Markets operated by people of color were more likely to have vendors who accept DUFB and SNAP than any other nutrition assistance programs. Vendors in people-of-color-led markets participated in these two programs at a higher rate than vendors at White-managed farmers markets. On the other hand, more vendors at White-managed farmers markets participate in WIC and Project FRESH than vendors at people-of-color-led markets. The age of the farmers market manager also matters. Markets operated by managers who were 55 years and older were much less likely to have vendors participating in DUFB, SNAP, WIC, and Project FRESH than markets operated by younger managers.

Michigan’s farmers markets are engaged in other activities that help to reduce food insecurity. Consequently, the study examined if vendors at farmers markets participated in seven types of food assistance activities (Table 4). Market managers were asked to say what vendors did with unsold food at the end of the market session. Vendors were most likely to donate unsold food to food pantries (77.6%) and sell it at reduced prices (79.6%). More than half of the vendors donated unsold food to soup kitchens, shelters, and churches.

**Table 4.** Farmers Market Vendors and Food Assistance Activities.

Food assistance activities	Total sample	Market location			Race/ethnicity of market managers		Gender of market managers		Age of primary market managers		
	Number of markets reporting	Rural	Urban clusters	Urbanized areas	White	People of color	Women	Men	Younger than 40 years	40 to 54 years	55 Years or older
Donate food to soup kitchens	51	12	14	25	46	5	40	11	15	20	15
No	49.0	91.7	57.1	24.0	47.8	60.0	47.5	54.5	46.7	55.0	46.7
Yes	51.0	8.3	42.9	76.0	52.2	40.0	52.5	45.5	53.3	45.0	53.3
Donate food to food pantries	58	14	15	29	52	6	45	13	18	20	19
No	22.4	42.9	26.7	10.3	21.2	33.3	26.7	7.7	27.8	15.0	26.3
Yes	77.6	57.1	73.3	89.7	78.8	66.7	73.3	92.3	72.2	85.0	73.7
Donate food to a shelter	52	13	14	25	47	5	41	11	16	20	16
No	38.5	61.5	57.1	16.0	40.4	20.0	39.0	36.4	37.5	40.0	37.5
Yes	61.5	38.5	42.9	84.0	59.6	80.0	61.0	63.6	62.5	60.0	62.5
Donate food to an emergency food aggregator	52	12	13	27	47	5	41	11	16	20	15
No	55.8	91.7	76.9	29.6	59.6	20.0	58.5	45.5	37.5	60.0	73.3
Yes	44.2	8.3	23.1	70.4	40.4	80.0	41.5	54.5	62.5	40.0	26.7
Donate food to schools	50	12	14	24	45	5	40	10	16	19	14
No	64.0	91.7	64.3	50.0	66.7	40.0	62.5	70.0	50.0	73.7	71.4
Yes	36.0	8.3	35.7	50.0	33.3	60.0	37.5	30.0	50.0	26.3	28.6
Donate food to religious institutions	52	14	14	24	46	6	41	11	16	20	15
No	46.2	57.1	64.3	29.2	50.0	16.7	48.8	36.4	50.0	40.0	46.7
Yes	53.8	42.9	35.7	70.8	50.0	83.3	51.2	63.6	50.0	60.0	53.3
Sell food at reduced prices	54	15	15	24	49	5	42	12	18	18	17
No	20.4	26.7	20.0	16.7	18.4	40.0	21.4	16.7	22.2	16.7	17.6
Yes	79.6	73.3	80.0	83.3	81.6	60.0	78.6	83.3	77.8	83.3	82.4

Vendors selling at markets in urbanized areas are far more likely to engage in food assistance activities than vendors in urban clusters or rural areas. For instance, 8.3% of vendors in rural markets donate unsold food to soup kitchens. In comparison, 42.9% of the vendors at markets in urban clusters and 76% in urbanized areas donate food to soup kitchens. In addition to soup kitchens, less than 10% of the vendors at rural markets donate unsold food to emergency food aggregators or schools. The data show that more than 70% of the vendors in urbanized markets donate unsold food to soup kitchens, food pantries, shelters, food aggregators, and religious institutions. At the end of the market session, more than 80% of the vendors at markets in urban areas also sold food at discounted prices.

Roughly 79% of the vendors in White-operated farmers markets donated unsold food to food pantries, and 81.6% sold the food at reduced prices. Similarly, 80% or more of the vendors in people-of-color-led markets donate food to shelters, emergency food aggregators, and churches at the end of the market session. Vendors at men-operated farmers markets were significantly more likely than those at women-operated markets to donate unsold food to food pantries. So, 92.3% of the vendors at men-operated markets and 73.3% of those with women managers donate unsold food to pantries.

Regardless of the age of the farmers market manager, similar percentages of the vendors in the markets they operate donate unsold food to shelters. The pattern does not hold for donations to food aggregators. Roughly 63% of the vendors in markets operated by the youngest managers donate unsold food to emergency food aggregators. This compares to only 26.7% of the vendors in markets operated by the oldest managers donating unsold food with aggregators and rescuers. Food aggregators such as Food Gatherers (2020) in Ann Arbor collect or rescue food from several locations, redistribute it to pantries, soup kitchens, shelters, serve it in their soup kitchens, and provide emergency groceries to those in need.

### Farmers Market Finances

Respondents were asked if the farmers market they managed lost money, broke even, or made a profit in 2019. All but one of the study participants responded to the question. Most of the farmers markets either broke even or made a profit in the year before the study. According to the managers, 38.9% of their markets made a profit, and 26.4% broke even. Roughly 14% of the markets lost money (Table 5).

**Table 5.** Farmers Markets Financial Outcomes—2019.

Market finances	Total sample	Market location			Race/ethnicity of market managers		Gender of market managers		Age of primary market managers		
	Number of markets reporting	Rural	Urban clusters	Urbanized areas	White	People of color	Women	Men	Younger than 40 years	40 to 54 years	55 Years or older
2019 Market financial outcomes	72	19	20	33	65	7	52	20	23	21	27
Percentage of markets that lost money	13.9	10.5	10.0	18.2	9.2	57.1	17.3	5.0	8.7	14.3	18.5
Percentage of markets that broke even	26.4	21.1	25.0	30.3	27.7	14.3	21.2	40.0	26.1	19.0	29.6
Percentage of markets that made a profit	38.9	57.9	35.0	30.3	41.5	14.3	38.5	40.0	30.4	38.1	48.1
Percent don’t know	20.8	10.5	30.0	21.2	21.5	14.3	23.1	15.0	34.8	28.6	3.7

Rural farmers markets are more likely than other markets to report that they made a profit in 2019. While 57.9% of the rural markets made a profit, only 35% of the markets in urban clusters, and 30.3% of those in urbanized areas made a profit. Roughly 10% of the rural markets and markets in urban clusters lost money; 18.2% of the markets in urbanized areas lost money.

There is a significant difference in the percentage of White-operated markets and people-of-color-managed markets that reported losing money in 2019. The study found that 9.2% of White-operated markets and 57.1% of the markets led by people of color lost money in the year before the study. Hence, while 41.5% of markets led by Whites made a profit, only 14.3% of the markets managed by people of color made a profit in the period studied.

Markets led by the oldest market managers had the highest likelihood of reporting a profit; 48.1% of these markets had a budgetary surplus in 2019. At the other end of the spectrum, markets run by the oldest market managers were also most likely to report that they lost money. Almost 9% of the markets operated by managers under 40 years of age lost money in 2019, but twice as many markets operated by managers who were 55 years and older lost money. That is, 18.5% of the markets operated by the oldest farmers market managers lost money.

Though similar percentages of men-operated and women-run farmers markets reported making a profit, men-operated markets are far less likely to report losing money than women-run markets. Men-operated markets are three-and-a-half times less likely to lose money than women-operated markets. Hence, 5% of the men-operated markets lost money compared with 17.3% of the women-managed markets.

### Impacts of the Coronavirus Pandemic (COVID-19) Market Operations

The story of the spread of the COVID-19 pandemic in Michigan is reflected in the reports obtained from Michigan’s farmers market managers about how their markets functioned in 2020. The outbreak started in urban centers such as Detroit first spread through the southern, most densely populated part of the state. Rural areas and the northern portion of the state had no or relatively few diagnosed cases of the virus till the middle of the summer (State of Michigan, 2020).

Spring farmers markets were halted, and the opening of summer markets delayed because of the pandemic. It was the case for 46.4% of the markets. The late start affected markets in urban clusters and urbanized areas much more than rural markets. While 17.6% of rural markets delayed their start, 56% of the nonrural markets had to postpone the beginning of their markets (Table 6).

**Table 6.** Farmers Markets and the Impacts of the Pandemic.

Impacts of the pandemic	Total sample	Market location		
	Number and percentage of markets reporting	Rural	Urban clusters	Urbanized areas
Effect on the start time of market activities	69	17	18	34
Started market activities later than usual	46.4	17.6	55.6	55.9
Started market activities the same time as usual or earlier	53.6	82.4	44.4	44.1
Effect on the size of market staff	70	17	18	35
Staff size decreased	22.9	11.8	22.2	28.6
Staff size remained the same	62.9	82.4	72.2	48.6
Staff size increased	14.2	5.8	5.6	22.8
Effect on the number of market volunteers	67	15	17	35
Number of volunteers has decreased	31.3	13.3	23.5	42.9
Number of volunteers remained the same	49.3	53.3	58.8	42.9
Number of volunteers has increased	19.4	33.4	17.7	14.2
Effect on the number of vendors	69	16	18	35
Number of vendors decreased	66.6	62.5	55.6	74.3
Number of vendors remained the same	11.6	12.5	11.1	11.4
Number of vendors increased	21.8	25.0	33.3	14.3
Effect on the number of customers	64	16	17	31
Number of customers decreased	43.8	31.3	41.2	51.6
Number of customers remained the same	15.6	12.5	17.6	16.1
Number of customers increased	40.6	56.2	41.2	32.3
Effect on the amount of people seeking food assistance from the market	65	17	18	30
The amount of people seeking food assistance has decreased	7.7	11.8	11.1	10.0
The amount of people seeking food assistance has remained the same	32.3	23.5	50.0	26.7
The amount of people seeking food assistance has increased	60.0	64.7	38.9	63.3
Effect on the amount of funding	67	15	18	34
The amount of funding has decreased	37.3	40.0	38.9	35.3
The amount of funding has remained the same	43.3	46.7	44.4	41.2
The amount of funding has increased	19.4	13.3	16.7	23.5
Effect on access to technical assistance	64	15	17	32
Access to technical assistance has decreased	26.2	33.3	29.4	21.9
Access to technical assistance has remained the same	67.2	60.0	58.8	75.0
Access to technical assistance has increased	6.6	6.7	11.8	3.1

Roughly 23% of the markets reported that the size of their staff shrunk. Again, rural markets were much less likely to report a reduction in staff than other markets. Markets in urban clusters and urbanized areas were about twice as likely to report staff contractions than rural markets. Markets in urbanized areas also reported increasing staff than other markets. So less than 6% of the rural markets and markets in urban clusters reported an increase in their staff, but 22.8% of the markets in urbanized areas said they increased their staff.

Rural markets were the least likely to report a decline in the number of volunteers. They were also most likely to report that the number of volunteers in their markets increased. The farmers markets in urbanized areas were the opposite. A high percent (42.9%) reported a reduction in the number of their volunteers, while only 14.2% said the number of their volunteers increased.

Across the state, the number of vendors in most farmers markets shrunk. The study found that two-thirds of the markets reported fewer vendors in 2020 than in previous years. Almost three-quarters of the markets in urbanized areas reported having fewer vendors. Markets in urbanized areas were also much less likely than other markets to say the number of vendors in their markets had increased.

Overall, 43.8% of the markets said the number of customers declined. While 31.3% of the rural markets indicated that their customer base shrunk, 51.6% of the urbanized markets reported fewer customers. In contrast, more than half of the rural farmers markets reported that the number of customers in their markets had increased. The number of people asking for food assistance from farmers markets increased during the pandemic. Sixty percent of the markets reported a surge in the number of people seeking food assistance. So, 64.7% of rural markets and 63.3% of markets in urbanized areas, and 38.9% of markets in urban clusters report increases in the request for food assistance from their markets.

At the same time, the demand for food assistance is rising at farmers markets, the amount of funding markets receive is declining or remaining stagnant. The data revealed that 37.3% of the markets had decreased funding; only 19.4% said they had increased funding in 2020. Farmers markets in urbanized areas were most likely to report that the amount of funding they secured had increased.

About two-thirds of the farmers markets reported that their access to technical assistance during the pandemic remained the same before the outbreak. About 26% of the markets reported a decline in access to technical assistance, while 6.6% said they had greater access to technical help since the pandemic broke out.

### Market Responses to the Pandemic

Some farmers markets responded to the pandemic by closing for the summer. Market managers explained how concern for their staff and vendors led to the closure of markets. Two respondents said,

Market operations [were] suspended this summer [because] most of the staff are older and more vulnerable to COVID . . . [The staff] have been making root beer and giving it to people to sell at other markets for next year’s budget.

… The market CLOSED—all activities were closed down for the summer. The farmers [who] were from vulnerable populations, decided to stay home. [They] did not feel safe in public. [We] . . . did not have enough farmers to run it [the market]. [We] . . . are making face masks now in one of [our] . . . sewing programs . . . [We] will be running in 2021.

Market managers report that market staff moved quickly to respond to the pandemic. As Table 7 shows, Michigan’s farmers markets employed various strategies to deal with the pandemic. Most frequently, signs asking customers to wear face coverings were posted in the markets; 37% of the markets adopted this strategy. Other strategies were adherence to local and state guidelines and practicing social distancing. A fifth of the markets reconfigured the layout of market stalls to create greater distance between vendors. Markets also installed hand sanitizing stations. Staff and vendors were required to wear masks in 16.7% of the markets. A manager reported, “One vendor dropped out because she refused to wear a mask.” However, market staff was able to enforce the mask-wearing policy with others.

**Table 7.** Market Responses to the Pandemic.

Responses^a^	Number of markets reporting	Percentage of cases (*n* = 54)
Post signs and pay for advertising to ask shoppers to wear masks	20	37.0
Follow MIFMA, local, Michigan, and Centers for Diseases Control (CDC) guidelines	12	22.2
Practice social distancing	12	22.2
Reconfigure market to get greater spacing between stalls	11	20.4
Provide hand sanitizing station	11	20.4
Require all market staff and vendors wore masks	9	16.7
Provide hand-washing station	8	14.8
Used arrows to designate one-way flow of foot traffic	7	13.0
Cancel summer market	6	11.1
Institute curb-side pickup for on-line orders; create pick-up days	6	11.1
Provide masks, paid for masks, etc. from market funds	6	11.1
Eliminate social activities in the market and cancel special events	6	11.1
Create online platform for vendors to sell products	5	9.3
Regulate and limit crowd size	5	9.3
Share guidelines, strategies and information with vendors and other market managers	5	9.3
Focus on staff and vendors because they are older; they are from vulnerable populations	4	7.4
Increase social media marketing; use Facebook to advertise; link to vendor’s Facebook pages	4	7.4
Cancel on-site cooking demonstrations, cancel educational events, and special markets	4	7.4
Write a pandemic preparedness plan and implement it	3	5.6
Switch to making products that could be sold instead of produce	2	3.7
Install plexi glass shields	2	3.7
Provide portable bathrooms	2	3.7
Switch to Community Supported Agriculture (CSA) model	2	3.7
Expand the physical size of the market to provide more space for vendors to socially distance	2	3.7
Make pre-sales available	2	3.7
Limit the number of vendor spaces	2	3.7
Wait for the government to provide guidance and help	2	3.7
Ask health-screening questions at entrance of markets	2	3.7
Vendors place protective table in front of their produce display stall to enforce distance	2	3.7
Eliminate food sampling	2	3.7
Cancel a part of the summer and winter markets	2	3.7
Create single stream of sales and limited-contact sales; limit exchange of currency	2	3.7
Distribute food assistance tokens, sanitize SNAP tokens	2	3.7
Create a perimeter to facilitate one-way traffic flow; limit the amount of parking	2	3.7
Clean more frequently	2	3.7
Sell at other markets that were open	1	1.9
Make face masks	1	1.9
Close bathrooms	1	1.9
Obtain foundation funding for pandemic response	1	1.9
Create a food hub for the farmers market	1	1.9
Use volunteers to assemble and box products for sale	1	1.9
Increase the number of volunteers	1	1.9
Change market venue to operate in a safer space	1	1.9
Limit the number of staff at the market	1	1.9
Add more staff	1	1.9
Assign personal shoppers to each customer to limit handling of products	1	1.9
Prepackage fruits and vegetables	1	1.9
Create walk-up window	1	1.9
Put mobile food truck in operation	1	1.9
Customers cannot touch the produce	1	1.9
Customers are not allowed to bring their own shopping bags	1	1.9
Reduce number of markets held each month	1	1.9
Distribute boxes of free produce to residents	1	1.9
Turn away vendors wanting to sell products that were not permitted	1	1.9
Food assistance redemption increased transaction fees	1	1.9

a Multiple responses—the percentages will total more than 100%.

Eleven percent of the markets switched to online ordering combined with curbside and no-contact pickup. Market managers said they increased the use of social media to advertise their market and encourage customers to shop. Some linked the market’s Facebook page to those of their vendors to promote the vendors and cultivate an atmosphere of online marketplaces. Managers reported that they,

Implemented an online order system, customers can order 7 days per week and there are 2 pickup days. 80% of sales acquired this way in April to May and now (July) has dropped to 60-70%.

Managers utilized another innovative strategy by adopting a commission model. The markets sold the products for vendors, and for this, the markets got a small commission. As one manager described the process, we

… created a single stream for sales by selling for our vendors at a 10% commission, which limited contact between people exchanging currencies and limited the number of workers at the market. Our setup was effectively modeled on old general stores, with personal shoppers assigned to each customer to limit interaction with products.

Markets also curtailed social activities like concerts, cooking demonstrations, and educational activities to reduce crowding and socializing. Managers were conflicted about reducing the social and community-building aspects of the markets, but ultimately decided that public safety was paramount in their operations.

Markets also distanced the customers from the vendors (by placing a table between the products displayed and the customer). Markets also enacted policies that prevented customers from handling products being sold or sampling food. Some markets sold prepackaged foods, while others had designated staff and volunteers shop with and for customers to limit contact with the vendors and the food. Managers also sanitized the tokens between use.

Some market managers expressed frustration with the overall government response to the pandemic. They felt they were left to develop responses independently or had to divert scarce resources to respond to the outbreak. Such managers said,

We are “praying that our federal government removes their head from their rear and designs a plan to confront this crisis. Because of the Wild West mentality of the gov’t, we took on all added PPE [personal protective equipment] expenses ourselves and policed the market for COVID protections in accordance with the _______ Health Department.”

The pandemic highlighted the vulnerability of campus-based farmers markets. These operate on several Michigan college campuses and college campuses elsewhere. When colleges and universities closed or went online, campus markets were also closed. One respondent wrote,

We are a university-based market, so we have not been able to open yet, the campus is closed to all faculty and students and we are facing budget cuts which may not make our market operational this season.

One farmers market focused on alleviating food insecurity in the market’s host neighborhood rather than opening and carrying on their usual activities. The market manager wrote,

Instead of having a traditional farmers market, we distributed free produce boxes provided to us by ______ Market. We distributed 75 boxes [in] . . . 6 weeks.

Another said,

We struggled to open, we struggled to keep operating at a level that made sense for our vendors. We had to spend funds intended for advertising and promotion on equipment to meet sanitation/protection standards. We had to turn vendors, and their stall fee revenues, away because they weren’t selling the permitted products. There were increased costs for us in the way of fees for processing increased food assistance transactions.

### Government Funding Assistance During the Pandemic

Eighty percent of the farmers market managers report that they have not received any government funding to help with the pandemic (Table 8). One manager said, we have received *“*Zero dollars, and zero help.” Another manager felt that farmers markets were overlooked in the distribution of aid. The manager lamented that “Farmers markets, in general, are low on their priority list so no surprise . . . [that aid] dollars are [going to] support . . . [the] unemployed and other core living requirements.”

**Table 8.** Government Funding or Assistance During the Pandemic.

Funding or assistance	Number of markets reporting	Percentage of cases (*n* = 60)
None	48	80.0
Received city funding	4	6.7
Received Economic Injury Disaster loan	2	3.3
Collect data on pandemic-related expenses; hoping for reimbursement	2	3.3
Paycheck Protection Program loan	2	3.3
Personal protective equipment	1	1.7
Received COVID rapid response funds	1	1.7

Only 6.7% of the markets received funding from cities and 3.3% received economic injury disaster loans or paycheck protection program loans. Another 3.3% are awaiting reimbursement for expenses they incurred for pandemic response.

### Feelings About the Government Responses to the Pandemic

Table 9 shows market managers’ positive and negative perceptions about government responses to the pandemic. Ten percent of the managers felt that MIFMA provided helpful resources during the pandemic. The managers said,

The real benefit to our market has been through the great efforts of MIFMA: through its webinars, its staff who have been extremely helpful! Having government funding for the increase in DUFB monies available has been very beneficial. But not allocating any further increase in Senior Project FRESH and WIC monies is truly lacking in scope . . . these recipients were/are in as much need as any other group of people.

I haven’t felt government engagement was great but MIFMA did a darn good job of getting information [to us that] we needed to run [the] market safely.

**Table 9.** Market Manager’s Perceptions of Government Responses to the Pandemic.

Responses^a^	Number of markets reporting	Percentage of cases (*n* = 60)
*Positive perceptions*		
The Michigan Farmers Market Association was helpful	6	10.0
Governor designated farmers markets as essential service	6	10.0
State government has been helpful	3	5.0
Local government has been helpful	3	5.0
Grateful that the state increased Bridge Card (SNAP) and Double-Up-Food Bucks monies	2	3.3
Happy the market was kept open	2	3.3
Government protected public health and safety	2	3.3
Pandemic precautions were easy to follow and had everyone’s interests in mind	1	1.7
Government assistance helped to build online platforms to distribute food	1	1.7
Happy to be able to operate the market	1	1.7
Farmers market coalition has been helpful	1	1.7
Small number of government grants	1	1.7
Happy to have a mask mandate in the markets	1	1.7
Had larger numbers of customers	1	1.7
Provided information on precautions to adopt	1	1.7
Received assistance	1	1.7
*Negative perceptions*		
No engagement or contact from the state or federal government	17	28.3
Confusing messaging from the state government	2	3.3
Lack of understanding about the importance of local foods	2	3.3
Did not get government assistance	2	3.3
Communication is challenging	2	3.3
SNAP recipients are not allowed to purchase food online	2	3.3
Disgusted and frustrated with response	2	3.3
Could not operate the market during the summer	1	1.7
Farmers market seem to be low on the priority list	1	1.7
Overlooking the Senior Project Fresh and WIC programs and not funding them	1	1.7
City government response was slow, hampered by city council voting and other red tape	1	1.7
Not allowing greenhouses to sell transplants was a mistake	1	1.7
Had to rely on the news to get information	1	1.7
Customers failing to follow government rules and mandates	1	1.7
Government not helping with the acquisition and purchase of personal protective equipment	1	1.7
Limited support for vendors – many lost money because of decreased sales	1	1.7
Could not run SNAP program because of uncertainty over opening market	1	1.7
Sanitizing SNAP tokens between each customer’s use is challenging	1	1.7
Farmers markets need more assistance	1	1.7
Restrictions placed on markets	1	1.7
Needs more guidance	1	1.7

a Multiple responses—the percentages will total more than 100%.

Managers were happy that the state’s governor declared that farmers markets were essential businesses, thereby paving the way for markets to open or stay open. They reported that the

State of Michigan government has been helpful. Sat in a committee that set guidelines for reopening agricultural activity. City government has been supportive, farmers market opened right after golf courses. No relationship [at] present with federal assistance.

Our local gov’t is great and has reached out with encouragement. Our state gov’t is broken, and the federal gov’t is in pieces . . . thus NO HELP!

Market managers expressed frustration with their perceived lack of engagement with the state and federal government; 28.3% of the managers indicated that this was their experience. They associate the lack of contact with the government with challenges they experience serving SNAP and DUFB customers. They identify and point to inequities that they want to reduce in their markets. The pandemic has exposed a problem in the processing of food assistance purchases. Recipients of these programs cannot use their EBT cards to make online purchases or place pre-orders. They made statements like

[I am] disappointed especially since SNAP sales can’t be offered online. It is unfair for SNAP customers to have low-contact pick-up instead of no-contact pick up.

… The inability to process SNAP and DUFB through our curbside pick-up system is disappointing . . . It restricts the number of customers that can utilize the online order—curbside pick-up option.

There really has not been a good amount of communication to the markets from the government, our market issues tokens for the government assistance programs and with the need to sterilize between patrons’ uses that would be a large hurdle for us to implement.

## Discussion

### Race, Gender, Age, Locality, and Farmers Market Dynamics

Our finding that 73.4% of the farmers market managers studied are women is consistent with earlier studies that found a high percentage of women occupying this position in the markets (e.g., see Mohammad et al., 2019; Smalley et al., 2017; C. L. Miller & McCole, 2014). However, our study is the first to note that the distribution of men and women managers is not evenly spread across locales. Urbanized areas and small urban clusters have a significantly higher percentage of women market managers than rural markets.

Our findings are also consistent with other research stating that the Michigan farmers’ market managers tend to be younger than other farmers in the state (C. L. Miller & McCole, 2014). However, our study reveals other new findings also. There is a gradient in the age distribution of farmers market managers that corresponds to locality. Hence, the mean age of market managers is highest in rural areas and lowest in urbanized areas. Like MIMFA (2017), we found that farmers market managers in our study were managers at their current market for less than five years. It raises questions about the experience level and longevity of the farmers market managers in the three localities and how age is correlated with market operations.

Earlier studies report that most farmers’ market vendors are White, and more than half are men (Campbell, 2014; S. M. Miller, 2005). Our study supports the finding on the racial/ethnic composition of the vendors, but not the gender. Our findings are more in line with Mohammad et al. (2019) who found that women vendors comprised 60% of their sample. Farmers market managers participating in our study reported that an average of 56.6.% of their market vendors were women.

Though Onianwa et al. (2006) report that less than half of the farmers market customers in their study was White, Elepu and Mazzocco (2009) indicate that more than 80% of their customers are White. Our findings are more consistent to the latter research finding, and we found that about three quarters of the customers in the markets we studied were White.

Several researchers found that 60% or more of the farmers market customers are women (Saddler et al., 2013; Byker et al., 2012; Baker, Hamshaw & Kolodinsky, 2009; Zepeda, 2009; Elepu & Mazzocco, 2009; Onianwa et al., 2006; Wolf, Spittler & Ahern, 2005). Our findings are analogous to these results.

Market size varied by locality. The markets in urbanized areas had the most vending spots, the highest weekly vendor occupancy rate, highest number of customers, and the longest waiting lists to get a booth in the market. The markets with the largest number of vendor spots, highest occupancy rates, and the most extended waiting lists were run by White managers. However, people-of-color-operated markets had more customers than markets operated by Whites.

Our finding of short job tenure among farmers market managers is consistent with the findings of other studies (MIFMA, 2017). It is a cause for concern that should be the subject of further investigation to find out the causes of job turnover among market managers. Is turnover due to low wages, meager or no benefits, the need for managers to have multiple jobs to make ends meet, or the lack of full-time employment? How is turnover related to infrastructure and support from local and state governments? Researchers should consider market saturation, the closure of some markets, and a market’s financial viability when assessing turnover among market managers.

Additionally, we should also find out if and how the tenure of market managers is related to the customer and vendor base. Though a change in a market’s management can allow new leaders and ideas to surface, frequent changes in managers can erode trust and depress the number of customers and vendors participating in the markets. Management changes can also affect food assistance programming.

Even though farmers market managers find it challenging to process food assistance benefits (Ming et al., 2019), many of Michigan’s farmers markets participate in programs to reduce food insecurity. We found that more than average Michigan farmers markets accept SNAP and WIC. Nationwide an average of 50.1% of the farmers markets process payments made through the nutrition described above assistance programs (USDA, 2020). Ninety percent of the Michigan farmers markets studied processed nutrition assistance program payments. Furthermore, more than 60% of the vendors in these markets participated in DUFB, SNAP, WIC, and Project FRESH.

The study found a relationship between the age of the farmers market manager and the market’s participation in nutrition assistance programs. The oldest farmers market managers operate markets that are less likely to participate in food assistance programs than other markets. The study also found that the age of the farmers market manager is also related to profitability. The markets with the oldest managers were most likely to report making a profit in 2019.

The youngest farmers market managers are making an impact worth noting. The youngest managers tend to operate in markets that have low vacancy rates and can attract large numbers of customers to their markets. It would behoove us to examine the markets more thoroughly to determine what practices and strategies are being used to make them profitable or attract large numbers of customers. The best practices identified can be applied elsewhere.

### A Snapshot of Farmers Market Managers of Color and the Markets They Operate

This study provides a snapshot of people of color farmers market managers that have not been described before. It is the case because studies of farmers market managers have not investigated market management by people of color. The findings of this study reveal that most people of color managers operate markets in urbanized areas. Moreover, the people of color managers tend to be younger than White managers. Again, the question of experience and its relation to market operations is pertinent.

People of color managers have been managing their current market for a mean of 2.7 years, and this length of tenure is shorter than it is for White managers. Policies and action plans should be put in place to help support and extend the tenure of market managers of color.

People of color managers operate small markets; these have about half the number of available vendor spots and a weekly vendor occupancy rate that is significantly lower than White-operated markets. Notwithstanding, people-of-color-led markets tend to have more customers than other markets. While people-of-color-managed markets have a mean of 228.3 customers weekly, White-operated markets have a mean of 214 customers per week.

Markets with people of color managers also have more people of color vendors and customers of color than White-operated markets. Michigan is a state where about 25% of the population are people of color (U.S. Census Bureau, 2020a). Nevertheless, more than a third of the customers at people-of-color-operated farmers markets are people of color. This finding is noteworthy as earlier studies have found that low numbers of people of color visit and shop at farmers markets (Nuss et al., 2017; Singleton et al., 2020; Skizim et al., 2017).

The larger number of customers in people-of-color-led markets may indicate greater customer demand for people-of-color-managed markets. If the demand is more significant for these types of markets, there should be greater effort to provide more opportunities for people of color to manage farmers markets. Those interested in broadening the appeal of farmers markets and attracting people of color to visit and shop in the markets should be cognizant of our finding that having a person of color at the helm of the market improves the chances of achieving those twin goals.

A higher percentage of the vendors in farmers markets managed by people of color participate in the DUFB, SNAP, Project FRESH, Hoop Houses for Health, Food Navigator, and Prescription for Health programs. The people-of-color-managed markets also participate in other activities that help reduce food insecurity. Vendors at these markets are more likely than vendors at White-operated markets to donate unsold food to shelters, emergency food aggregators, schools, and churches.

Though the people of color markets have been operating in their current locations for about eight years, they manifest signs of financial instability. Fifty-seven percent of the markets managed by people of color lost money in 2019. The people-of-color- led farmers markets are about six times more likely to report losing money than White-operated markets. The pandemic made people-of-color-led markets more vulnerable, placing additional financial stresses on these markets.

### Farmers Markets and the Pandemic

Markets in urbanized areas were heavily affected by the pandemic early on, and the impacts continued through the summer and fall. Farmers markets in urban clusters and rural areas felt the pandemic’s effect from the middle of the summer onwards. The pandemic affected market staff, volunteers, vendors, and customers. Some vendors were reluctant to participate in markets because they felt they would contract the virus. At the same time, customers shunned the markets as they avoided crowded venues.

The pandemic resulted in a spike in food donations from farmers markets, coupled with a decline in the amount of funding available to markets. Managers also report an increase in the number of nutrition assistance recipients patronizing farmers markets. However, farmers market managers complained that they encountered unexpected problems in processing EBT payments online. The current EBT system does not allow food assistance program participants to make purchases online or place preorders. It placed food assistance recipients at a disadvantage and exposed them to unnecessary risk because they still had to visit the markets in person to make purchases. The inequity created by the processing problem meant that food assistance recipients could not opt for no-contact purchases at farmers markets.

Some markets reported that they experienced a decline in access to the technology needed to run the markets. The pandemic essentially crippled one type of market—campus farmers markets. During the second half of the spring and throughout the summer, college campuses across the state closed their doors to students, faculty, and staff. That left campus farmers markets with no one to operate, sell, or buy goods in them.

## Conclusions

This study challenges researchers to think more deeply about the concept of farmers markets as White spaces and devise studies to interrogate the idea systematically. It urges scholars to examine more than a handful of farmers markets to understand better how racialization manifests itself in different markets. In addition to race/ethnicity, the study also demonstrates how locality, age, gender, market manager characteristics, vendor characteristics, and customer characteristics are related to market appearance, culture and operation.

More financial training and safety nets are needed for market managers, especially for the newest and youngest managers. The markets need financial support. More people turned to farmers markets for food assistance during the pandemic, yet the markets received paltry amounts of government financial support. Though farmers markets were essential services, farmers markets were left on their own to respond to the pandemic and fill the increased demand for food donations. It is challenging for farmers market vendors as many are small farmers who barely break even or operate on small profit margins.

MIFMA and other agencies such as the state’s Department of Health and Human Services, the USDA should conduct further studies to see if these findings are generalizable to Michigan and beyond. USDA should take note of the disadvantages low-income customers faced when they could not make online purchases with EBT cards during the pandemic and ensure that poor customers do not have to take undue risks to obtain food in the future.
